# 1843. Exploring Cisgender Women's HIV Pre-Exposure Prophylaxis (PrEP) Needs and Preferences Across Settings: the Roles of Social-Structural Factors

**DOI:** 10.1093/ofid/ofad500.1671

**Published:** 2023-11-27

**Authors:** Deanna Kerrigan, Kimi Yonamine, Allison O’Rourke, Tahilin Sanchez Karver, Wendy W Davis, Aimee A Metzner, Alan Oglesby, Cindy Garris, Rachel Scott, Patricia Moriarty, Tamara Taggart, Clare Barrington, Hoisex Gomez, Martha Perez, Yeycy Donastorg

**Affiliations:** George Washington University, Washington, District of Columbia; The George Washington University, Herndon, Virginia; George Washington University, Washington, District of Columbia; Johns Hopkins Bloomberg School of Public Health, Baltimore, Maryland; George Washington University Milken Institute School of Public Health, Washington, District of Columbia; ViiV Healthcare, Durham, North Carolina; ViiV Healthcare, Durham, North Carolina; ViiV Healthcare, Durham, North Carolina; MedStar Health Research Institute, Washington, DC; MedStar Health Research Institute, Washington, DC; George Washington University, Washington, District of Columbia; UNC Gillings School of Global Public Health, Chapel Hill, North Carolina; Unidad de Vacunas e Investigación IDCP, Santo Domingo, Distrito Nacional, Dominican Republic; Instituto Dermatologico y Cirugia de la Piel, Santo Domingo, Distrito Nacional, Dominican Republic; Instituto Dermatologico y Cirugia de Piel "Dr. Huberto Bogaert Diaz", Santo Domingo, Distrito Nacional, Dominican Republic

## Abstract

**Background:**

Cisgender women (CGW) account for 48% of new HIV infections globally and 18% of new HIV infections in the United States (US) but comprise only 8% of daily oral PrEP users in the US. Social-structural factors including stigma, gender norms, and medical provider bias have been linked to daily oral PrEP inequities among CGW. These factors influencing PrEP utilization merit further attention as additional options emerge, including long-acting (LA) injectable PrEP.

**Methods:**

PrEP preferences, barriers, and facilitators to uptake were explored through 60 in-depth interviews with CGW with indications for PrEP (n=40), and HIV and reproductive healthcare providers (HCPs) (n=20) in Washington, DC, US and Santo Domingo, Dominican Republic (DR) in 2021 and 2022 (**Table 1**). CGW in the DR were female sex workers (FSW), while those in DC were seeking reproductive health services. Thematic content analysis was employed.

**Figure:**
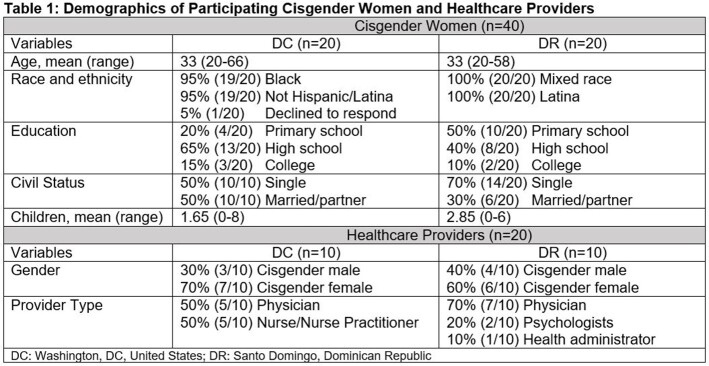

**Results:**

Participants noted low awareness of PrEP, particularly LA PrEP, among CGW across settings and indicated this may be in part due to ongoing emphasis on PrEP for men who have sex with men and transgender women compared to CGW. In DC and the DR, PrEP was often seen as a potential empowerment tool by CGW to address social-structural constraints, gender-related pressures and responsibilities, and inequitable partner dynamics. The perceived need for and preferences regarding daily oral vs. LA injectable PrEP was mixed among CGW in DC due to varied self-assessment regarding PrEP as a priority and some CGW’s comfort with pill taking. LA PrEP was described by some CGW as a means to improve adherence and reduce stigma associated with pill taking. FSW in the DR were particularly interested in LA PrEP as they found it more compatible with their work. Suggested facilitators of PrEP uptake noted across settings included financial and logistical support (transport, childcare) and HCP training on CGW’s PrEP needs.

**Conclusion:**

Tailored CGW-centered awareness campaigns and sensitivity training for HCPs related to PrEP options for CGW are needed. Access to LA PrEP for FSW is critical given the disproportionate impact of HIV on this community and their work-related PrEP preferences. Interventions must address social-structural factors among CGW to support equitable PrEP uptake.

**Disclosures:**

**Aimee A. Metzner, PharmD, AAHIVP**, ViiV Healthcare: Full-time employee (salary/benefits/etc.)|ViiV Healthcare: Stocks/Bonds **Alan Oglesby, MPH**, GlaxoSmithKline: Employment|GlaxoSmithKline: Stocks/Bonds **Cindy Garris, MS**, GSK: Stocks/Bonds|ViiV Healthcare: Employee **Rachel Scott, MD,MPH,FACOG**, Gilead Science: Grant/Research Support|ViiV/GSK: Grant/Research Support

